# Purpose in Life and Cognitive Function: Evidence for Momentary Associations in Daily Life

**DOI:** 10.1093/geroni/igae018

**Published:** 2024-02-15

**Authors:** Angelina R Sutin, Martina Luchetti, Alyssa A Gamaldo, Jacqueline Mogle, Hephzibah H Lovett, Justin Brown, Martin J Sliwinski, Antonio Terracciano

**Affiliations:** Department of Behavioral Sciences and Social Medicine, Florida State University College of Medicine, Tallahassee, Florida, USA; Department of Behavioral Sciences and Social Medicine, Florida State University College of Medicine, Tallahassee, Florida, USA; Department of Psychology, Clemson University, Clemson, South Carolina, USA; Department of Psychology, Clemson University, Clemson, South Carolina, USA; Department of Behavioral Sciences and Social Medicine, Florida State University College of Medicine, Tallahassee, Florida, USA; Department of Behavioral Sciences and Social Medicine, Florida State University College of Medicine, Tallahassee, Florida, USA; Human Development and Family Studies, The Pennsylvania State University, State College, Pennsylvania, USA; Department of Geriatrics, Florida State University College of Medicine, Tallahassee, Florida, USA

**Keywords:** Ecological momentary assessment, Eudaimonic well-being, Hedonic affect, Processing speed

## Abstract

**Background and Objectives:**

Purpose in life is associated with healthier cognitive outcomes in older adulthood. This research examines within-person dynamics between momentary purpose and cognitive function to provide proof of concept that increases in purpose are associated with better cognitive performance.

**Research Design and Methods:**

Participants (*N* = 303; 54% female; *M*_age_ = 51.71, *SD* = 7.32) completed smartphone-based momentary assessments of purpose and short cognitive tasks 3 times a day for 8 days.

**Results:**

In moments when participants felt more purpose driven than their average, they had faster processing speed (*b* = −1.240, *SE* = 0.194; *p* < .001), independent of person, temporal, and contextual factors and practice effects. Momentary purpose was unrelated to visual working memory performance (*b* = −0.001, *SE* = 0.001; *p* = .475). In contrast to purpose, momentary hedonic affect (e.g., happiness) was unrelated to momentary cognition.

**Discussion and Implications:**

Feeling more momentary purpose may support faster processing speed in daily life. Such evidence provides stage 0 support for a purpose-based intervention for healthier cognition, which may be particularly useful in middle adulthood and the transition to older adulthood before the onset of cognitive impairment.

**Translational Significance:** Purpose in life is an aspect of eudaimonic well-being that is associated consistently with healthier cognitive function. This research shrinks the timescale to examine whether momentary, naturally occurring feelings of purpose are associated with better cognitive performance in daily life. Middle-aged adults completed surveys and brief cognitive tasks on a smartphone three times a day for eight days. In moments when participants felt more purposeful than their average, they had faster processing speed. These findings support further work on purpose as a promising target of intervention for healthier cognitive aging.

## Background and Objectives

Purpose in life, the feeling that one’s life is goal-oriented and driven (Ryff, 1995), has been associated consistently with better cognitive health (Sutin, Luchetti, & Terracciano, 2021). Individuals who report more purpose, for example, tend to perform better on tasks of memory and verbal fluency (Sutin, Luchetti, Stephan, et al., 2022), maintain their cognitive function longer into older adulthood (Kim et al., 2019), and are less likely to develop pre-dementia syndromes that increase risk of dementia (Sutin et al., 2018; Sutin, Luchetti, Stephan, et al., 2021). This healthier cognitive profile culminates in lower risk of incident dementia: Higher purpose in life among individuals with healthy cognitive function is associated with lower risk of subsequently developing dementia over time, an association that has replicated in at least eight samples (Sutin, Luchetti, Aschwanden, et al., 2023). This association may be due, in part, to the healthier lifestyles and clinical profiles associated with purpose: Individuals higher in purpose tend to engage in more physical activity (Hooker & Masters, 2016), are less likely to use substances (Kim et al., 2020), are more socially integrated (Sutin, Luchetti, Aschwanden, et al., 2022), and less likely to live with chronic diseases (Musich et al., 2018). These factors account for some, but not all, of the association between purpose and healthier cognitive outcomes (Boyle et al., 2010). Greater engagement in everyday life may also be an additional mechanism in the pathway between purpose and cognitive function (Sutin, Luchetti, Stephan, et al., 2023).

There is evidence that purpose in life can be increased through intervention (Manco & Hamby, 2021; Park et al., 2019), which makes it a particularly promising target for healthier cognitive outcomes. Yet, more stage 0 evidence (Onken et al., 2014) is needed to further support moving to intervention development. Although the emerging literature provides consistent evidence for the positive association between purpose and better cognitive function, the evidence thus far has been from cognitive assessments that have taken place either at a single point in time or measured repeatedly with long intervals in between assessments. Cognitive function, however, is dynamic and fluctuates across the span of a single day (Cerino et al., 2021; Nicosia et al., 2023; Weizenbaum et al., 2020), and there are reliable and valid measures to assess momentary cognitive function (Sliwinski et al., 2018). Indeed, a growing literature indicates that cognitive performance does vary across the day (Campbell et al., 2020; Nicosia et al., 2023), is sensitive to cognitive impairment (Cerino et al., 2021), and that transitory factors are associated with such fluctuations (Hawks et al., 2023; Whibley et al., 2022; Zhaoyang et al., 2021). Momentary fluctuations in purpose in life have yet to be examined as a predictor of momentary cognitive performance.

Although purpose is typically conceptualized as a relatively stable aspect of well-being (i.e., an enduring tendency rather than sensitive to acute fluctuations; Hill & Weston, 2019; McKnight & Kashdan, 2009), recent evidence suggests that there is also a state component to it. One study, for example, assessed purpose in life monthly and found that about 30% of its variance was attributable to within-person fluctuations (Pfund, DeLongis, et al., 2022). A second study assessed purpose each evening and found that about 40% of the variance was attributable to within-person fluctuations (Pfund, Hofer, et al., 2022). This variance suggests there might be significant intraindividual fluctuations in purpose over short periods of time. To our knowledge, no study has addressed moment-to-moment variability measured within days, which is a prerequisite for associations with momentary cognitive function.

The present research examines within-person associations between momentary purpose and cognitive function in everyday life. We expect momentary purpose and cognitive function to fluctuate throughout the day and hypothesize that in moments when individuals feel more purposeful than their average, they will have better momentary cognition. In addition, we address whether these associations are specific to momentary purpose or extend to hedonic affect. Adults in middle age and early older adulthood were the focal population for this research because this period of adulthood is critical for supporting cognitive health to prevent cognitive impairment in older adulthood (Livingston et al., 2020).

## Research Design and Methods

### Participants and Procedure

Participants were from the Couples Healthy Aging Project (CHAP). The purpose of CHAP was to examine factors related to cognitive health in everyday life. Participants were recruited as couples. Inclusion criteria were that participants had to be in a committed relationship for at least 1 year, both members of the couple had to be between the ages of 40 and 70, the couple had to cohabitate, and both members of the couple had to be willing to participate. There was no exclusion based on marital status or sexual orientation. The protocol was approved by the institutional review board at the Florida State University (#STUDY00000472) and was carried out in accordance with the Declaration of Helsinki. All participants provided informed consent before testing.

Participants completed a virtual assessment over Zoom with study staff, a survey online through Qualtrics, and an eight-day ecological momentary assessment (EMA). The EMA assessment was on a study-provided Android phone that participants carried around over the course of the 8 days. All participants used the same type of device provided by the study for the momentary assessments, and the phone was not connected to cellular service or Wi-Fi and was thus not dependent on a reliable connection. Participants were trained on the EMA portion of the study during their baseline interview before the start of the eight days of assessment. During the EMA portion of the study, participants were alerted at three semirandom times throughout the day to rate their beliefs and feelings in the moment and to complete several short cognitive tasks (see below); they also completed a short nightly questionnaire that asked general questions about their day. A total of 308 participants enrolled in the study. Of these participants, 303 had data from the EMA assessment; missing EMA data (*n* = 5) were due to technical issues with retrieving data from the phones. On average, participants completed 91% (29 of 32 total assessments) of the EMA/nightly assessments over the 8 days.

### Measures

#### Momentary purpose and hedonic affect

Momentary purpose was measured with the item, “How PURPOSE-DRIVEN do you feel right now?” This item was the focal item to examine the association between momentary purpose and cognition. Hedonic affect was measured with the items “How HAPPY do you feel right now?” and “How much ENJOYMENT/FUN do you feel right now?” For each item, participants responded on a slider that ranged from 0 (not at all) to 100 (extremely). The two hedonic items were averaged within each momentary assessment as a measure of hedonic affect.

#### Momentary cognition

Participants completed two short cognitive tasks: symbol search and dot memory (Sliwinski et al., 2018). The symbol search task was used to measure processing speed. On each trial, participants saw a row of two symbol pairs at the top of the screen and two symbol pairs at the bottom of the screen. Participants had to decide, as quickly as possible, which of the two pairs at the bottom of the screen matched one of the symbol pairs at the top of the screen. Participants completed 12 trials of the task at each momentary assessment. The dot memory task measured visual working memory. Participants saw a 5 × 5 checkerboard with three red dots at specific locations around the screen. They had 3 s to study the dot locations and were then directed to complete a filler task (tapping all the fs on a screen of fs and es for 8 s). Following the filler task, participants were directed to a blank checkerboard and were asked to indicate the location of the red dots. Participants completed two test trials. The focal outcome for symbol search was mean reaction time for correct responses across the 12 trials at each momentary assessment (incorrect responses were not included in the mean reaction time) and the focal outcome for dot memory was the Euclidean distance (or error distance) from the placed dot to the correct location of each dot (correct placement received a score of 0), averaged across trials within each momentary assessment. As such, for both tasks, lower scores indicated better performance (faster reaction time and fewer errors, respectively).

#### Trait purpose

Trait purpose in life was assessed with a 7-item version of the purpose subscale from the Ryff Measures of Psychological Well-being (Ryff, 1989) administered as part of the baseline survey. Participants rated items (e.g., “I have a sense of direction and purpose in my life.”) on a scale from 1 (*strongly disagree*) to 5 (*strongly agree*). Items were reverse scored when necessary and the mean taken in the direction of greater purpose in life (alpha = .72).

#### Covariates

Sociodemographic covariates were age in years, sex (1 = female, 0 = male), race (1 = person of color, 0 = White), and education in years. Temporal and contextual covariates were day in the study when each assessment was completed (range 1–8), time window (1 = morning, 2 = midday, 3 = afternoon), weekday (= 1) versus weekend (= 0), location (1 = work, 0 = other location), and presence of other person(s) at the time of assessment (1 = yes, 0 = no).

### Analytical Strategy

Multilevel linear models were used to account for the nested data structure: measurement occasions (Level 1) were nested within individuals (Level 2). A third level was tested to account for participants nested within couples (Level 3), but because the results were the same, we report the two-level model. First, we assessed the association between momentary purpose and performance on each cognitive task. Model 1 included a person-mean-centered score for momentary purpose as a predictor of momentary cognitive performance. This person-mean-centered score reflected participants’ deviations from their mean level of purpose across assessments. That is, the participant mean level of momentary purpose was taken across assessments and subtracted from each momentary score. We further accounted for average momentary purpose derived across momentary assessments (Model 1.1) and trait purpose assessed in the baseline survey (Model 1.2). Model 2 extended Model 1 to account for person factors (age, sex, race, education). Model 3 accounted for temporal and contextual factors (study day, time window, weekday versus weekend day, location, and presence of other persons at the time of assessment) in addition to person factors. To account for practice effects, we further controlled for the total number of assessments completed during the study period by each participant; we entered a linear and quadratic term as predictor of cognitive performance. All models allowed for a random intercept; all continuous Level 2 (=individual) variables were grand mean centered.

We then examined whether the associations extended to hedonic emotions (mean of momentary feeling happy and enjoyment/fun). For this supplementary analysis, we estimated the basic (Model 1) and fully adjusted model (Model 3).

## Results

Descriptive statistics for all study variables are in Table 1. Correlations among study variables are in Supplementary Table 1. Across the 303 participants, there were 6,579 momentary assessments over the eight-day period with a mean of 21.7 (*SD* = 2.9) assessments per participant (range = 7 to 24). There was significant within- and between-person variance in momentary purpose across these assessments: 65% of the variance in momentary purpose was within person, which indicated sufficient variability that could be associated with other momentary variables. As expected, there was also sufficient within-person variance for processing speed (35% for reaction time) and dot memory (73% for distance errors).

**Table 1.** Descriptive Statistics for Study Variables at the Participant Level

Variable	*M* (*SD*)	% (*n*)	ICC	1-ICC
Age (years)	51.71 (7.32)		—	—
Sex (female)		54.5 (165)	—	—
Education (years)	16.59 (3.32)		—	—
Race (people of color)		24.1 (73)	—	—
Geographic region (United States)		100.0 (303)		
Trait purpose	4.21 (0.59)		—	—
Momentary measures^a^				
Purpose	69.13 (15.79)		0.349	0.651
Hedonic emotions	63.58 (15.18)		0.399	0.601
Symbol search, mean reaction time	1,615.10 (44.71)		0.652	0.348
Dot memory dot, mean distance error	1.45 (0.73)		0.266	0.734

*Notes: N* = 303. ICC = intraclass correlation coefficient. ICCs show the proportion of variance in a measure attributed to between-person variability. Thus, 1-ICC provides the proportion of variability that can be attributed to within-person variability.

^a^Mean calculated across momentary assessments.

In the basic model for symbol search (Table 2; Model 1), there was the expected association between momentary purpose and processing speed: In moments when participants felt more purpose-driven than their average across assessments, they performed better (faster) on the symbol search task (the association was the same adjusting for task accuracy). Controlling for average levels of momentary purpose (Model 1.1) or trait purpose (Model 1.2; i.e., between-person purpose) did not account for this association (Supplementary Table 2). The association also remained significant controlling for person factors (Table 2; Model 2), temporal and contextual factors (Model 3), and practice effects (Supplementary Table 3). Momentary purpose was unrelated to performance on the dot memory task across all models (Table 2). There was also no cross-level interaction between momentary purpose and average levels of purpose for either outcome.

**Table 2.** Momentary Association Between Purpose and Symbol Search (Mean Reaction Time) and Dot Memory (Mean Distance Error)

Variable	Model 1			Model 2			Model 3		
	*B*	SE	*p* Value	*B*	SE	*p* Value	*B*	SE	*p* Value
*Mean reaction time*									
Intercept	1,614.965	25.327	<.001	1,634.760	36.821	<.001	1,751.030	39.358	<.001
**Momentary purpose**	−1.369	0.194	<.001	−1.370	0.194	<.001	−1.240	0.194	<.001
Level 2 covariates (between-person)									
Age				22.360	3.151	<.001	22.418	3.144	<.001
Sex (female)				−120.030	46.016	.010	−121.175	45.915	.009
Race (people of color)				189.380	53.711	<.001	189.470	53.587	<.001
Education				−5.086	6.899	.462	−5.224	6.884	.449
Level 1 covariates (within-person)									
Day in the study							−27.426	1.735	<.001
Time window							1.263	4.793	.792
Location (work)							−19.591	11.206	.080
Weekend day							−17.245	9.494	.069
With others							21.908	8.866	.014
*Variance components*									
Residual	99,881.514	1,791.758	<.001	99,885.416	1,791.827	<.001	95,764.571	1,718.180	<.001
Intercept	188,332.324	15,738.377	<.001	149,761.683	12,594.242	<.001	149,238.259	12,535.749	<.001
*Mean distance error*									
Intercept	1.451	0.042	<.001	1.196	0.064	<.001	1.233	0.082	<.001
**Momentary purpose**	−0.001	0.001	.216	−0.001	0.001	.219	−0.001	0.001	.475
Level 2 covariates (between-person)									
Age				0.025	0.005	<.001	0.025	0.005	<.001
Sex (female)				0.345	0.080	<.001	0.343	0.080	<.001
Race (people of color)				0.294	0.093	.002	0.294	0.093	.002
Education				−0.029	0.012	.017	−0.028	0.012	.018
Level 1 covariates (within-person)									
Day in the study							−0.048	0.006	<.001
Time window							0.077	0.018	<.001
Location (work)							−0.022	0.041	.581
Weekend day							−0.021	0.035	.548
With others							0.062	0.033	.055
*Variance components*									
Residual	1.300	0.024	<.001	1.300	0.024	<.001	1.283	0.023	<.001
Intercept	0.472	0.044	<.001	0.396	0.038	<.001	0.394	0.037	<.001

*Note:* Momentary purpose was person-mean centered. The score reflects participants’ deviations from their mean level of purpose across assessments. The participant mean level of momentary purpose was taken across assessments and subtracted from each momentary score. Level 2 continuous variables are grand mean centered.

In contrast to momentary purpose, momentary hedonic affect was unrelated to symbol search performance as well as dot memory performance (Supplementary Table 4), suggesting that better processing speed is associated with feelings of being more purposeful in the moment rather than feelings of happiness and enjoyment in the moment.

## Discussion and Implications

The present research took a novel approach to the association between purpose in life and cognitive function. Building on the growing literature that a greater sense of purpose in life supports healthier cognitive aging (Sutin, Luchetti, & Terracciano, 2021), the present research brought this association to the momentary level and found that in moments when individuals felt more purpose-driven than their average, their processing speed was faster. Momentary purpose was unrelated to visual working memory. The association between momentary purpose and processing speed was robust to person, temporal, and contextual factors and practice effects. This momentary association was also limited to purpose-related feelings rather than well-being more broadly.

Cognitive function has been traditionally measured in lab settings or over long periods of time. There are also, however, meaningful variations in cognitive function across the day that can be captured with EMA (Sliwinski et al., 2018). And, indeed, in the present sample, there was considerable within-person variability in momentary cognitive performance. The current EMA methodology also demonstrated that purpose fluctuates even across the span of a single day. Recent research has highlighted the within person variability in feelings of purpose measured daily (Pfund, Hofer, et al., 2022) and weekly (Pfund, DeLongis, et al., 2022). The present research extended this variability to within-day, momentary fluctuations. Such variability was a prerequisite for momentary associations with cognitive function. And, consistent with our expectations, we found an association between momentary purpose and processing speed. In moments when participants felt more purposeful than their average, they had faster processing speed. This finding provides new evidence for the association between purpose and cognition at the momentary level. Previous research has primarily focused on between-person cross-sectional associations (e.g., purpose is associated with episodic memory and verbal fluency; Sutin, Luchetti, Stephan, et al., 2022) or longitudinal associations over years (e.g., purpose tends to be associated with less cognitive decline over time in older adulthood; Kim et al., 2019). This beneficial association extends to within-person fluctuations in processing speed throughout the day.

The present findings contribute to theoretical models of purpose and cognitive health. Purpose in life has been recognized as a component of well-being that supports health across adulthood (McKnight & Kashdan, 2009). Purpose is thought to contribute to better health-related outcomes in part through greater engagement (Ryff et al., 2016). Faster processing speed could be a marker of engagement that helps keep the brain active. Over time, this activity may protect the brain from neuropathology. Such engagement in everyday life may support the maintenance of brain structures and functions and increase resistance against neurodegeneration (Nyberg et al., 2012). Purpose may also help build greater reserve and increase resilience to withstand neuropathology and support better cognitive function into older adulthood (Stern et al., 2020). The faster processing speed may support these mechanisms in the pathway from purpose to healthier cognitive outcomes.

The well-being literature makes the distinction between eudaimonic well-being that reflects feelings of living a meaningful life and hedonic well-being that reflects pleasurable emotional states (Ryan & Deci, 2001). Eudaimonic aspects of well-being have been associated consistently with cognitive function (Sutin, Luchetti, Stephan, et al., 2022), whereas the association between hedonic aspects of well-being and cognition has been more mixed (Berk et al., 2017; Castro-Schilo et al., 2019; Zhu et al., 2023). At the momentary level, hedonic affect is unrelated to cognitive performance (Daniëls et al., 2020; Hawks et al., 2023; Small et al., 2019). Previous studies of momentary cognition, however, have not measured momentary feelings of eudaimonic well-being. The present findings are consistent with the small literature on momentary hedonic affect and cognition and highlight the importance of momentary eudaimonic well-being for momentary performance. Feeling momentarily purposeful may help focus attentional resources needed to perform well on the processing speed task; feelings of positive affect may not recruit such resources.

It is important to note that there was no relation between either momentary purpose or momentary hedonic affect and performance on the visual working memory task. Although this task has been validated (Sliwinski et al., 2018), it tends not to be associated with other psychosocial and contextual factors that vary across moments (Zhaoyang et al., 2021). The task may not be able to capture momentary variations in this cognitive function or working memory itself may be more stable than processing speed (Cerino et al., 2021) and thus more resistant to momentary fluctuations in psychological factors. It is also possible that the processes necessary to perform well on visual working memory tasks may be unrelated to purpose. Successful performance on this task requires the integration of several cognitive processes, including attention, working memory, and visuospatial ability (Miyake et al., 2001). It may be that purpose is associated with basic cognitive processes but not with tasks that require the more complex integration of processes. Processing speed, in contrast, may be more reactive to momentary fluctuations (Small et al., 2019; Zhaoyang et al., 2021). Given that intraindividual variation in cognitive function (Gamaldo et al., 2012), particularly processing speed (Haynes et al., 2017), is associated with an increased risk of dementia, it is a critical cognitive function to track over time. Support for better processing speed may help to maintain better overall cognitive function and support healthier cognitive aging for longer into older adulthood.

Purpose in life can increase through intervention (Manco & Hamby, 2021; Park et al., 2019), which makes it a promising intervention target because there is already evidence for its malleability. The present research provides proof of concept that when individuals feel more purposeful than their average, they have better cognitive performance in the moment. The next step for intervention development is to experimentally increase purpose to determine its causal effect on cognitive performance. Such a mechanism-based approach would provide evidence for how an intervention leads to better cognitive outcomes (Nielsen et al., 2018). A reminiscence intervention, for example, may increase purpose in life (Pinquart & Forstmeier, 2012). Specifically, in a reminiscence intervention, individuals are led to think about the important and meaningful experiences in their lives, both positive and negative, and to evaluate and integrate these experiences into a larger, more coherent narrative of one’s life (Westerhof & Slatman, 2019). If a reminiscence intervention increases purpose, and this increase in purpose supports better cognitive function, an increase in purpose may be the mechanism of action that leads to better cognitive function over time.

The sample for the current research focused on middle-aged adults. This focus was strategic given the importance of midlife health for cognitive outcomes in older adulthood, which has been emphasized in Alzheimer’s disease and related disorders prevention efforts (Livingston et al., 2020). It is also a period of the lifespan somewhat overlooked in the literature on both purpose and cognition, where the focus tends to be on development and the transition to adulthood on one side of the lifespan and late life aging and related outcomes on the other side. Greater support for purpose in life in middle adulthood may have cascading benefits into older adulthood. Future research needs to better address the long-term outcomes of midlife purpose for late-life cognition. Future research is also needed to identify sociocontextual factors that support feeling more purposeful in the moment (e.g., social support), particularly in this period of the lifespan (midlife), where many transitions occur that could affect feelings of purpose.

The present study had several strengths, including a relatively large sample for an EMA study, brief cognitive assessments three times a day for 8 days, and the momentary assessment of both eudaimonic and hedonic aspects of well-being. There are also some limitations. First, the focus on middle age may limit generalizability to other age groups. There may be differences in cognitive processes in both younger and older adults that shape the association with momentary purpose. The sample was also primarily White and educated; future research needs to extend these associations in samples with other sociodemographic characteristics to determine generalizability (e.g., the association between purpose in life and less cognitive decline has been found to be stronger among Black adults; Kim et al., 2019). Second, the design was correlational rather than experimental. As such, although it was apparent that higher momentary purpose relative to one’s average was associated with faster processing speed, this association is based on observations of variation in daily life, rather than experimental manipulations of purpose, and could also be interpreted in the other direction (i.e., in moments when individuals have faster processing, they feel more purpose driven). Future research would benefit from experimental paradigms to provide stronger evidence that purpose supports better cognitive function. Third, the momentary hedonic items measured valence but not arousal. There could be some element of arousal or alertness responsible for both momentary feelings of purpose and better cognitive performance (and/or could be the mechanism for the association). Future research could measure arousal to better identify the momentary relations between purpose and cognition. Finally, the measurement-burst design provided short-term intensive repeated-measures data, but longitudinal data would be informative for better addressing the mechanisms through which purpose leads to long-term cognitive outcomes.

Despite these limitations, the present research provides insight into the dynamics of momentary purpose and cognitive function in daily life. The current findings indicate that greater feelings of being purpose-driven relative to one’s average in the moment are associated with better processing speed. These findings provide additional stage 0 evidence that supports further work to develop purpose-based interventions to support healthier cognitive aging starting at least as early as middle age.

## Supplementary Material

igae018_suppl_Supplementary_Table_S1-S4

## Data Availability

This research was not preregistered. Materials, data, and syntax for the current study are available at https://osf.io/74y9d/?view_only=8ba06e9fa07c4c87ae97a56843e2a369. All measures administered in the parent study can be found at https://osf.io/74y9d/?view_only=8ba06e9fa07c4c87ae97a56843e2a369.
