# Supplementary material for: Purpose in Life and Cognitive Function: Evidence for Momentary Associations in Daily Life
Source: Innov Aging. 2024 Feb 15;8(3):igae018. doi: 10.1093/geroni/igae018 (PMC10953619; doi:10.1093/geroni/igae018)
Supplement: igae018_suppl_Supplementary_Table_S1-S4 [file igae018_suppl_supplementary_table_s1-s4.docx]

*Innovation in Aging* Supplementary Material: Sutin, Angelina R.; Luchetti, Martina; Gamaldo, Alyssa A.; Mogle, Jacqueline; Lovett, Hephzibah H.; Brown, Justin; Sliwinski, Martin J.; Terracciano, Antonio. Purpose in life and cognitive function: Evidence for momentary associations in daily life.

Supplemental Table S1

*Bivariate Correlations for Study Variables*

| Variables | 1 | 2 | 3 | 4 | 5 | 6 | 7 | 8 | 9 |
| --- | --- | --- | --- | --- | --- | --- | --- | --- | --- |
| 1. Age | 1 | -.106 | -.117* | -.104 | .140* | -.010 | .221** | .374** | .216** |
| 2. Sex |  | 1 | .102 | 0.058 | -.030 | .100 | -.046 | -.190** | .189** |
| 3. Education |  |  | 1 | -.054 | -.024 | .043 | -.108 | -.103 | -.150** |
| 4. Race |  |  |  | 1 | .112 | .032 | .028 | .128* | .133* |
| 5. Purpose, Momentary |  |  |  |  | 1 | .337** | .699** | .069 | .040 |
| 6. Purpose, Baseline |  |  |  |  |  | 1 | .263** | .039 | .061 |
| 7. Hedonic Emotions, Momentary |  |  |  |  |  |  | 1 | .087 | .047 |
| 8. Symbol Search, M Reaction Time |  |  |  |  |  |  |  | 1 | .253** |
| 9. Dot Memory, M Distance Error |  |  |  |  |  |  |  |  | 1 |

*Note.* Person’s correlations are reported for continues variables and Spearman’s rho are reported for categorical variables. We correlated person-mean scores; for each momentary measure, the mean was taken across all momentary assessments.

Supplemental Table S2

*Momentary Purpose and Trait Purpose*

| Symbol Search: | Model 1.1 |  |  | Model 1.2 |  |  |
| --- | --- | --- | --- | --- | --- | --- |
| *Mean Reaction Time* | B | SE | *p* | B | SE | *p* |
| Intercept | 1614.733 | 25.266 | <.001 | 1615.017 | 25.309 | <.001 |
| **Momentary Purpose (Level 1)** | -1.369 | 0.194 | <.001 | -1.369 | 0.194 | <.001 |
| **Between Person Purpose (Level 2)** | 1.932 | 1.606 | .230 | -- | -- |  |
| **Purpose Baseline Survey (Level 2)** | -- | -- |  | 28.701 | 42.814 | .503 |
| *Variance Components* |  |  |  |  |  |  |
| Residual | 99881.892 | 1791.764 | <.001 | 99881.212 | 1791.752 | <.001 |
| Intercept | 187395.763 | 15662.034 | <.001 | 188054.026 | 15715.690 | <.001 |
| Memory Dot: | Model 1.1 |  |  | Model 1.2 |  |  |
| *Mean Distance Error* | B | SE | *p* | B | SE | *p* |
| Intercept | 1.451 | 0.042 | <.001 | 1.451 | 0.042 | <.001 |
| **Momentary Purpose (Level 1)** | -0.001 | 0.001 | .216 | -0.001 | 0.001 | .215 |
| **Between Person Purpose (Level 2)** | 0.002 | 0.003 | .479 | -- | -- |  |
| **Purpose Baseline Survey (Level 2)** | -- | -- |  | 0.075 | 0.072 | .294 |
| *Variance Components* |  |  |  |  |  |  |
| Residual | 1.300 | 0.024 | <.001 | 1.300 | 0.024 | <.001 |
| Intercept | 0.471 | 0.044 | <.001 | 0.470 | 0.044 | <.001 |

*Note.* Level 1 continuous variables are person-mean centered. Level 2 continuous variable are grand mean centered.

Supplemental Table S3

*Accounting for Practice Effects*

|  | *Symbol Search: Mean Reaction Time* | | | *Memory Dot: Mean Distance Error* | | |
| --- | --- | --- | --- | --- | --- | --- |
|  | B | SE | *p* | B | SE | *p* |
| Intercept | 1762.039 | 40.723 | <.001 | 1.208 | 0.085 | <.001 |
| **Momentary Purpose** | -1.240 | 0.194 | <.001 | -0.001 | 0.001 | .473 |
| Level 2 Covariates  (between-person) |  |  |  |  |  |  |
| Age | 22.848 | 3.145 | <.001 | 0.025 | 0.005 | <.001 |
| Sex (female) | -119.276 | 45.764 | .010 | 0.348 | 0.080 | <.001 |
| Race (people of color) | 186.018 | 53.275 | .001 | 0.297 | 0.093 | .002 |
| Education | -6.140 | 6.874 | .372 | -0.029 | 0.012 | .017 |
| No. assessments | -16.767 | 8.464 | .049 | 0.009 | 0.015 | .548 |
| No. assessments (squared) | -1.088 | 0.944 | .250 | 0.002 | 0.002 | .247 |
| Level 1 Covariates  (within-person) |  |  |  |  |  |  |
| Day in the study | -27.415 | 1.735 | .000 | -0.048 | 0.006 | <.001 |
| Time window | 1.274 | 4.793 | .790 | 0.077 | 0.018 | <.001 |
| Location (work) | -19.573 | 11.207 | .081 | -0.021 | 0.041 | .604 |
| Weekend day | -17.201 | 9.495 | .070 | -0.020 | 0.035 | .562 |
| With others | 21.876 | 8.866 | .014 | 0.062 | 0.033 | .056 |
| *Variance Components* |  |  |  |  |  |  |
| Residual | 95763.242 | 1718.157 | <.001 | 1.283 | 0.023 | <.001 |
| Intercept | 147288.813 | 12376.831 | <.001 | 0.392 | 0.037 | <.001 |

*Note.* Level 1 continuous variables are person-mean centered. Level 2 continuous variable are grand mean centered.

Supplemental Table S4

*Momentary Association between Hedonic Affect and Symbol Search and Dot Memory*

| *Symbol Search:* | Model 1 |  |  | Model 3 |  |  |
| --- | --- | --- | --- | --- | --- | --- |
| *Mean Reaction Time* | B | SE | *p* | B | SE | *p* |
| Intercept | 1614.970 | 25.326 | <.001 | 1751.954 | 39.431 | 0.000 |
| **Momentary Hedonic Affect** | -0.062 | 0.224 | .781 | -0.304 | 0.231 | 0.187 |
| Level 2 Covariates  (between-person) |  |  |  |  |  |  |
| Age |  |  |  | 22.375 | 3.144 | 0.000 |
| Sex (female) |  |  |  | -121.921 | 45.919 | 0.008 |
| Race (people of color) |  |  |  | 189.153 | 53.592 | 0.000 |
| Education |  |  |  | -5.325 | 6.884 | 0.440 |
| Level 1 Covariates  (within-person) |  |  |  |  |  |  |
| Day in the study |  |  |  | -27.487 | 1.741 | 0.000 |
| Time window |  |  |  | 2.562 | 4.844 | 0.597 |
| Location (work) |  |  |  | -32.752 | 11.164 | 0.003 |
| Weekend day |  |  |  | -14.183 | 9.593 | 0.139 |
| With others |  |  |  | 21.162 | 8.987 | 0.019 |
| *Variance Components* |  |  |  |  |  |  |
| Residual | 100678.550 | 1806.055 | <.001 | 96368.403 | 1729.014 | <.001 |
| Intercept | 188275.235 | 15736.806 | <.001 | 149236.949 | 12537.977 | <.001 |
| *Dot Memory:* |  |  |  |  |  |  |
| *Mean Errors* | B | SE | *p* | B | SE | *p* |
| Intercept | 1.451 | 0.042 | <.001 | 1.233 | 0.082 | 0.000 |
| **Momentary Hedonic Affect** | 0.001 | 0.001 | .281 | -0.000 | 0.001 | 0.909 |
| Level 2 Covariates  (between-person) |  |  |  |  |  |  |
| Age |  |  |  | 0.025 | 0.005 | 0.000 |
| Sex (female) |  |  |  | 0.342 | 0.080 | 0.000 |
| Race (people of color) |  |  |  | 0.294 | 0.093 | 0.002 |
| Education |  |  |  | -0.028 | 0.012 | 0.017 |
| Level 1 Covariates  (within-person) |  |  |  |  |  |  |
| Day in the study |  |  |  | -0.048 | 0.006 | 0.000 |
| Time window |  |  |  | 0.077 | 0.018 | 0.000 |
| Location (work) |  |  |  | -0.028 | 0.041 | 0.496 |
| Weekend day |  |  |  | -0.020 | 0.035 | 0.574 |
| With others |  |  |  | 0.062 | 0.033 | 0.060 |
| *Variance Components* |  |  |  |  |  |  |
| Residual | 1.300 | 0.024 | <.001 | 1.283 | 0.023 | <.001 |
| Intercept | 0.472 | 0.044 | <.001 | 0.394 | 0.037 | <.001 |

*Note.* Level 1 continuous variables are person-mean centered. Level 2 continuous variable are grand mean centered.
